# Urinary sodium excretion and the risk of CVD: a community-based cohort study in Taiwan

**DOI:** 10.1017/S0007114521001768

**Published:** 2022-04-14

**Authors:** Yi-Jie Wang, Kuo-Liong Chien, Hsiu-Ching Hsu, Hung-Ju Lin, Ta-Chen Su, Ming-Fong Chen, Yuan-Teh Lee

**Affiliations:** 1 Institute of Epidemiology and Preventive Medicine, College of Public Health, National Taiwan University, No.17, Xu-Zhou Rd., Taipei City, 10055, Taiwan; 2 Department of Internal Medicine, National Taiwan University Hospital, No. 7, Zhongshan S. Rd., Zhongzheng Dist., Taipei City, 10002, Taiwan

**Keywords:** CVD, Urinary sodium excretion, Mediation analysis

## Abstract

Urinary Na excretion is a potential risk factor for CVD. However, the underlying biological mechanisms and effects of salt sensitivity are unclear. The purpose of this study was to characterise the relative contribution of biological factors to the Na–CVD association. A total of 2112 participants were enrolled in this study. Structured questionnaires and blood and urine samples were obtained. Twenty-four-hour Na excretion was estimated using a single overnight urine sample. Hypertension, the metabolic syndrome and overweight status were considered to indicate salt sensitivity. Cox proportional hazard models were used to investigate the effects of salt sensitivity on urinary Na excretion and CVD risk. The traditional mediation approach was used to calculate the proportion of mediation. The mean age (sd) of the 2112 participants was 54·5 (sd 12·2) years, and they were followed up for a mean of 14·1 (sd 8·1) years. Compared with those in the lowest quartile, the highest baseline urinary Na excretion (>4·2 g/24 h) was associated with a 43 % higher CVD risk (hazard ratio, 1·43; 95 % CI 1·02, 1·99). Participants with high urinary Na excretion, hypertension or the metabolic syndrome had a significantly high risk of CVD. The carotid intima-media thickness had the largest mediating effect (accounting for 35 % of the Na–CVD association), followed by systolic blood pressure (BP) (33 %), left ventricular mass (28 %) and diastolic BP (14 %). Higher urinary Na excretion increased the risk of CVD, which was explained largely by carotid media-thickness and systolic BP.

CVD, including CHD and stroke, is a major leading cause of global mortality^(1)^. Practically, 50 % of the non-communicable disease-related deaths were attributed to CVD^(2)^. The modifiable risk factors of CVD included unhealthy dietary habits, physical inactivity and smoking^(3)^. Previous studies found that high Na consumption was associated with CVD risk in both high-*v*.-low and dose–response meta-analyses^(4)^. The WHO recommends that dietary Na intake should be <2 g/d^(5)^. The American Heart Association has suggested that dietary Na intake should be reduced to <1500 mg/d^(6)^. Other institutions have endorsed a daily Na intake of no more than 2300 mg^(7–9)^.

High Na intake is associated with the risk of CVD^(10,11)^. A study suggested that low Na excretion is associated with high CVD mortality^(12)^. On the other hand, Lamelas *et al.* found a possible J-shaped association between 24-h Na excretion and CVD outcomes^(13)^. A few studies have indicated a non-significant relationship between dietary Na intake and CVD risk^(14,15)^. Previous studies have inconsistent results between Na intake and stroke or total CVD. In addition, the mediating effects of the Na–CVD association are not fully understood. Mediation analysis linking Na intake and CVD risk is necessary.

In nutritional epidemiology, both urinary Na excretion and dietary assessment tools are widely used to evaluate dietary Na intake. For urine collection, spot, overnight and 24-h urinary Na excretion are the usual methods for assessing Na intake. Twenty-four-hour urine collection is widely thought of as the valid method for assessment of Na intake because approximately 90 % of Na intake is excreted in the urine^(16)^. Multiple 24-h urinary Na excretion has been regarded as the gold standard for Na assessment despite its high burden and high cost^(17)^. On the other hand, the use of spot urine and overnight urine collection to estimate dietary Na intake is simpler and has been investigated for its feasibility^(18,19)^. In addition, because the plasma electrolyte concentration is homoeostatic, plasma Na concentration cannot reflect the amount of Na intake.

The heterogeneity of the blood pressure (BP) response to alterations in dietary Na intake between individuals has been found to affect the Na–CVD relationship^(20)^. In fact, the relationship between high Na intake and elevated BP varies between salt-sensitive individuals and salt-resistant individuals. By definition, salt sensitivity is an increase in BP of at least 10 % during a high-salt diet compared with a low-salt diet^(20)^. The predisposing factors of salt sensitivity include BMI and co-morbidities, such as hypertension and the metabolic syndrome^(20,21)^. Morimoto *et al.* conducted a 7·3-year follow-up study, which indicated that both fatal and non-fatal CVD events were twice as frequent in salt-sensitive individuals than in salt-resistant individuals^(22)^. Individual variation attributed to salt sensitivity has been found in the relationship between dietary Na intake and both elevated BP and increased CVD risk.

In this study, we aimed to investigate the association between urinary Na excretion and CVD risk, to explore the effects of salt sensitivity predisposing factors between urinary Na excretion and CVD risk and to quantify the contribution of novel biological factors to the Na–CVD association.

## Methods

### Study design and population

Study participants were enrolled in the Chin-Shan Community Cardiovascular Cohort Study (CCCC) in 1990 comprising 1703 men and 1899 women of Chinese ethnicity (response rate 82·8 %) and aged 35 years and older from the Chin-Shan Township, Taipei, Taiwan. The details of the CCCC study have been published^(23–25)^. We assessed information about anthropometry, lifestyle and medical conditions using interview questionnaires every 2 years for the initial 6 years. The validity and reliability of the collected data and measurements were previously reported in detail^(24)^. Briefly, we used a structured questionnaire to interview all participants in the baseline survey. To obtain data on demographic characteristics, lifestyle behaviours, regular exercise and personal and family history of diseases and hospitalisations, trained medical students investigated door-to-door with the help of community leaders to extend invitations for the baseline survey. The questionnaires were collected over the 2-year survey duration. Physicians and medical students performed physical examinations and laboratory tests for the participants who were invited to the clinic with their consent. The baseline CVD history and incident CVD cases were identified and established through questionnaires.

The inclusion criteria were participants without a CVD history at baseline and the first follow-up. Patients with incomplete urine samples were excluded from the analysis. The follow-up time was from 1992 to 2013, as the urine samples were collected at the first follow-up. A total of 2112 participants were included; of the participants excluded from the study, 887 had incomplete urine samples or did not submit their urine samples, 115 had baseline CVD and 488 were lost to follow-up. In the CCCC study, the participants were not representative of the general population because of the volunteer-based recruitment strategy and the participants were only from one community. The National Taiwan University Hospital Committee Review Board approved this study protocol. This study was conducted in accordance with the guidelines laid down in the Declaration of Helsinki, and all procedures involving human subjects were approved by the National Taiwan University Hospital (NTUH 105-S3120). Written informed consent was obtained from all participants.

### Exposure assessments

All participants were given a plastic container, taught how to collect their overnight urine samples and recorded their sleep duration. The participants were instructed to collect overnight urine after they excreted urine before sleeping. We recorded the sleep time and calculated the 24-h urine amount from sleep time and morning voiding urine^(18)^. The 24-h urine Na and excretion were calculated using the following formula: the overnight urine Na concentration multiplied by the overnight urine volume, divided by sleeping hours and then multiplied by 24. To obtain the usual Na excretion amount, the participants were asked not to change their dietary patterns when collecting overnight urine. Hence, the collection could be perceived as a marker of usual Na intake. Urinary electrolytes were measured using selective electrodes of the Dimension autoanalyzer (Du Pont)^(26,27)^. All urine samples not shipped at 4°C to the National Taiwan University Hospital clinic laboratory within 8 h for analysis were stored at –76°C using liquid N_2_.

### Biochemical measurements

A mercury sphygmomanometer was used to measure BP on the right arm twice, with the participants seated comfortably and arms supported and positioned at the level of the heart. The physicians utilised a 12-lead electrocardiogram for each participant and blindly evaluated the results. The average BP measurements were used, and the details of the average BP have been published previously^(28,29)^. BMI was calculated as weight (in kg)/height^2^ (in m^2^)^(30)^. After 12-h overnight fasting, venous blood samples were collected from all participants, refrigerated and transported within 6 h to the National Taiwan University Hospital. Then, the blood samples were stored at –76°C. Blood samples were collected at baseline (1990–1991), then at the first (1992–1993) and fourth (1997–1998) follow-up period. Plasma uric acid concentration was measured using commercial kits (Merck Chemical) and placed in an Eppendorf 5060 autoanalyzer (Eppendorf Corp)^(31)^. We used the Modification of Diet in Renal Disease Study equation to compute for the estimated glomerular filtration rate (eGFR) by serum creatinine, age, sex and body size^(32)^. Standard enzymatic tests were used to assess serum cholesterol, TAG and fasting glucose levels (Merck)^(33–35)^. The HDL-cholesterol levels were measured in the supernatants after the precipitation of specimens with magnesium chloride phosphotungstate reagents (Merck 14993)^(36)^. The LDL-cholesterol was calculated as the total cholesterol minus cholesterol in the supernatant using the precipitation method (Merck 14992)^(24)^. The carotid intima-media thickness was measured using a Hewlett-Packard SONO 1500 ultrasound system, equipped with a 7·5 MHz real-time B-mode scanner. The participants were required to lie on their back with their neck extended in a slightly lateral rotation. The carotid intima-media thickness was defined as the distance from the lumen-intima interface, indicated by the front edge of the first echogenic line, to the media-adventitia interface in the far wall of the vessel, indicated the front edge of the second line. The same procedure was performed on the other side of the neck. The maximum carotid intima-media thickness was identified by the average maximal measurement on both sides of the neck^(37)^. In the 1992–1993 follow-up period, standard M-mode echocardiographic measurements were conducted according to the recommendations of the American Society of Echocardiography to calculate the left ventricular mass using the modified American Society of Echocardiography method^(38)^.

### Clinical disease ascertainment

Hypertension was defined as systolic BP (SBP) of ≥140 mmHg and/or diastolic BP (DBP) of ≥90 mmHg and/or hypertensive therapy^(39)^. Overweight was defined as a BMI of ≥ 23·0 kg/m^2^ for both men and women; non-overweight was defined as a BMI of <23·0 kg/m^2^. This cut-off has been proposed by Japan, Asia-Oceania and WHO^(40,41)^. Participants with fasting glucose levels of ≥7 mmol/L, with the use of oral hypoglycaemic medications or insulin, were diagnosed as having diabetes. We defined the metabolic syndrome using the modified Asian adult treatment panel III criteria^(42)^. Participants with at least three of the following criteria were defined as having the metabolic syndrome: (1) BP of at least 130/85 mmHg or treated hypertension; (2) serum TAG level of at least 150 mg/dl; (3) HDL-cholesterol of <40 mg/dl in men and <50 mg/dl in women; (4) fasting glucose of ≥100 mg/dl or treated diabetes and (5) central obesity, defined as having waist circumference > 90 cm in men and >80 cm in women. Dysglycaemia was defined as fasting glucose of ≥100 mg/dl according to the modified Asian American Heart Association/National Heart, Lung, and Blood Institute Scientific Statement criteria^(43)^. Central obesity was defined according to the criteria of the Asian International Diabetes Federation^(44)^. As a consequence of no waist circumference data collected at baseline (1990–1991) for the CCCC study, we used the second follow-up waist circumference data (1994–1995) for baseline analysis.

### Endpoints ascertainment

Incident CVD events, including CHD and stroke, were reviewed by the physicians of the study team. To determine whether each event met the study protocol, four physicians reviewed the questionnaires, medical records, death certificates and laboratory data. CHD events were defined as coronary death, non-fatal myocardial infarction or hospitalisation due to procedures of the coronary artery bypass graft and percutaneous coronary intervention. Stroke events, including cerebral infarction and cerebral haemorrhage, were defined as death from different types of stroke or hospitalisation due to a sudden neurological deficit of vascular origin.

The cardiologists conducted conferences to clarify the causes of events without knowledge of the status of the participants. For the non-respondents, relatives of the uncooperative participants were contacted to obtain information on their health status. Medical records were examined at the Chin-Shan Community Health Center and National Taiwan University Hospital. The diagnostic criteria for CVD were based on the recommendations of the New York Heart Association and the American Heart Association^(45)^. The details of the incident CVD case ascertainment were reviewed previously^(28,29)^.

### Statistical analyses

Statistical analyses were performed using SAS software (version 9.4; SAS Institute) and R software version 3.6.1. The sample size was calculated using the methods mentioned in previous studies^(46,47)^. All statistical tests were two-tailed with a type I error of 0·05, and the statistical significance was set to *P* < 0·05. The participants were categorised based on quartiles of 24-h urinary Na excretion. We assumed the CVD event rate for participants in the low urine Na excretion group was 0·06. To conclude that participants in the high urine Na excretion group have higher risk of events than those in the low urine Na excretion group (hazard ratio (HR) = 1·8), at least 1764 participants were required to be recruited to achieve a power of 80 % at a significance level of 0·05.

Continuous variables are presented as mean and standard deviation, and ANOVA was used to test the differences across the quartiles. Categorical variables are presented and tested using the *χ* ^2^ test. Correlations between urinary Na excretion and other CVD risk factors were estimated using the Spearman’s partial correlation coefficients. The incidence rates of CVD, stroke and CHD cases were calculated by dividing the number of cases and person-years of follow-up for each quartile of 24-h urinary Na excretion. Cox proportional hazards models were used to adjust for potential confounders. The HR of CVD, CHD and stroke consisting of cerebral infarction and cerebral haemorrhage were calculated by dividing the incidence rate of urinary Na excretion in each quartile. Participants with the lowest urinary Na excretion levels were defined as the reference category. The median values of each urinary Na excretion group were used to assess linear trends (*P*_for trend_). Model 1 adjusted for age (35–44, 45–54, 55–64, 65–74 and ≥75 years of age) and sex. Model 2 additionally adjusted for lifestyle factors, including BMI (<18, 18–20·9, 21–22·9, 23–24·9 or ≥25 kg/m^2^), smoking (yes/no or abstinence), current alcohol drinking (regular/no), marital status (single, married and living with a spouse, or divorced or separated), regular exercise habits (yes/no), education level (≤9 years), occupation (unemployed, labourer, office worker or business person) and baseline hypertension (yes/no). Model 3 further included diabetes status (yes/no), LDL-cholesterol (mg/dl) and eGFR (<60, ≥60 ml/min per 1·73 m^2^). We performed linear and nonlinear relationships between urinary Na excretion and the risk of CVD, stroke and CHD. An ANOVA test was conducted to test the linearity assumption. Subgroup analysis was conducted and stratified by age (<65/≥65 years), sex, eGFR (<60/≥60 ml/min per 1·73 m^2^) and menopause status. To investigate the effects of hypertension, overweight status and the metabolic syndrome on the association between urinary Na excretion and CVD, stroke and CHD risks, we stratified the participants based on urinary Na excretion (<2/≥2 g/24 h). Then, the participants were further stratified by hypertension, the metabolic syndrome or overweight status. The HR of CVD, stroke and CHD were analysed according to the incidence rate in each group. The group in which Na excretion was <2 g/d and without hypertension, the metabolic syndrome or overweight status was considered as the reference group.

The Baron and Kenny approach was used to examine whether SBP, DBP, carotid intima-media thickness and left ventricular mass satisfied the criteria to be used as potential mediators based on the *a priori* hypothesis^(48)^. The four-step approach was used to establish a mediation relationship^(48)^. We performed the traditional mediation approach by comparing two regression models, one with conditioning and the other without on each mediator. First, we examined the significance of the association between urinary Na excretion and CVD risk in the basic model adjusted for age, sex, BMI, smoking status, current alcohol drinking, marital status, regular exercise habits, education level, occupation, LDL-cholesterol, eGFR and diabetes status. Then, we added each potential mediator one at a time to the basic model and examined the magnitude of each of the factors potentially mediating the association of urinary Na excretion with CVD risk. Next, the magnitude of the change in the HR was assessed for the highest *v*. the lowest urinary Na excretion group with and without adjustment for each potential mediator. The change in HR towards the null suggests a potential mediator effect on the urinary Na excretion-associated reduction in CVD risk. The proportion of CVD risk reduction explained by each mediator was calculated using the following formula: (HR_basic model_ − HR_adjusted model_)/(HR_basic model_ − 1) × 100 %^(49)^. In addition, we used the counterfactual framework approach as the sensitivity analysis^(50,51)^.

## Results

### Baseline characteristics

A total of 2112 participants comprising 975 men and 1137 women met all of the study criteria (online Supplementary Fig. S1). The mean age was 54·0 years, and the median follow-up period was 19·1 (interquartile range = 7·4–21·4 years). The participants in the highest quartile of urinary Na excretion had a higher education level, BMI, SBP, DBP, serum cholesterol, serum HDL-cholesterol, urinary uric acid, urinary potassium excretion, urinary salt excretion, estimated urine creatinine, overnight urine volume, and estimated 24-h urine volume, and had less sleep time. (Table 1).

**Table 1.** Baseline characteristics of study participants by urinary sodium excretion quartiles (Numbers; mean values and standard deviations)

	Q1		Q2		Q3		Q4		*P*
Range, g/24 h	< 2·0		2·0–2·9		2·9–4·2		> 4·2		
Median, g/24 h	1·5		2·4		3·5		5·3		
Number	528		528		528		528		
Gender (%)									0·58
Women	51·7		53·4		54·4		55·9		
Men	48·3		46·6		45·6		44·1		
Current smoker (%)	36·6		36·6		33·3		32·0		0·29
Alcohol drinking (%)	29·4		29·6		29·0		25·8		0·48
Married status (%)									0·94
Single	3·0		2·5		2·1		2·7		
Lived with a spouse	87·1		88·0		89·5		88·1		
Divorced or separate	9·9		9·5		8·4		9·3		
Education level (%)									0·04
<9 years	94·1		94·7		95·6		91·7		
≥9 years	5·9		5·3		4·4		8·3		
Job status (%)									0·22
No job	60·4		49·5		48·7		47·5		
Blue collar	33·3		35·6		33·7		31·4		
White collar	16·3		14·8		17·6		21·0		
Regular exercise habit (%)	14·4		16·1		14·8		16·5		0·75
Family history of CHD (%)	11·4		12·9		9·3		9·7		0·21
Family history of stroke (%)	21·0		19·9		23·1		20·3		0·58
Hypertension (%)	14·7		13·4		13·6		19·7		0·11
Diabetes mellitus (%)	13·5		13·4		14·0		17·0		0·30

	Mean	sd	Mean	sd	Mean	sd	Mean	sd	
Age (years)	54·0	12·7	55·0	11·9	53·8	11·4	53·2	10·8	0·10
BMI (kg/m^2^)	23·1	3·2	23·2	3·3	23·8	3·1	24·1	3·8	< 0·001
SBP (mmHg)	125·4	19·5	123·4	19·6	124·7	20·7	128·3	20·1	< 0·001
DBP (mmHg)	76·0	11·8	74·7	11·6	76·0	11·9	77·8	12·2	< 0·001
Total cholesterol (mg/dl)	194·3	43·9	201·4	45·3	200·4	44·1	201·3	46·2	0·03
TAG (mg/dl)	119·4	81·0	128·4	120·9	118·3	98·2	127·3	90·3	0·23
HDL-cholesterol (mg/dl)	48·0	16·0	50·0	15·5	49·4	14·5	50·6	15·3	0·03
LDL-cholesterol (mg/dl)	123·6	44·9	120·4	43·5	122·5	44·0	120·8	44·5	0·62
Fasting glucose (mg/dl)	110·3	29·8	111·0	32·7	111·2	38·0	115·5	36·9	0·07
Serum creatinine (mg/dl)	0·9	0·5	0·9	0·4	0·8	0·2	0·9	0·3	0·60
Glomerular filtration rate (ml/min per 1·73 m^2^)	96·7	99·9	100·0	74·2	95·8	44·7	98·2	49·7	0·78
Uric acid (mg/dl)	6·1	1·8	5·8	1·7	5·8	1·7	5·7	1·8	0·01
Urine sodium (mmol/24 h)	60·6	17·4	103·8	11·4	150·8	16·3	253·1	73·2	< 0·001
Urine potassium (mmol/24 h)	20·1	10·1	24·3	13·6	28·3	12·5	36·8	19·5	< 0·001
Urine sodium (g/24 h)	1·4	0·4	2·4	0·3	3·5	0·4	5·8	1·7	< 0·001
Urine salt (g/24 h)	2·4	0·7	4·1	0·5	6·0	0·6	10·0	2·9	< 0·001
Urine potassium (g/24 h)	0·7	0·4	0·9	0·5	1·1	0·5	1·4	0·8	< 0·001
Estimated urine creatinine (mg/24 h)	1269·1	231·3	1294·7	240·0	1324·3	240·3	1332·1	250·1	< 0·001
Overnight urine volume (ml)	263·2	113·5	303·5	132·7	339·7	143·2	461·9	253·7	< 0·001
Estimated 24-h urine volume (ml)	873·2	400·0	1027·8	462·9	1191·0	502·7	1654·4	641·6	< 0·001
Sleep time (h)	7·4	1·4	7·2	1·4	7·0	1·4	6·7	1·4	< 0·001

DBP, diastolic blood pressure; SBP, systolic blood pressure.

**Fig. 1.**
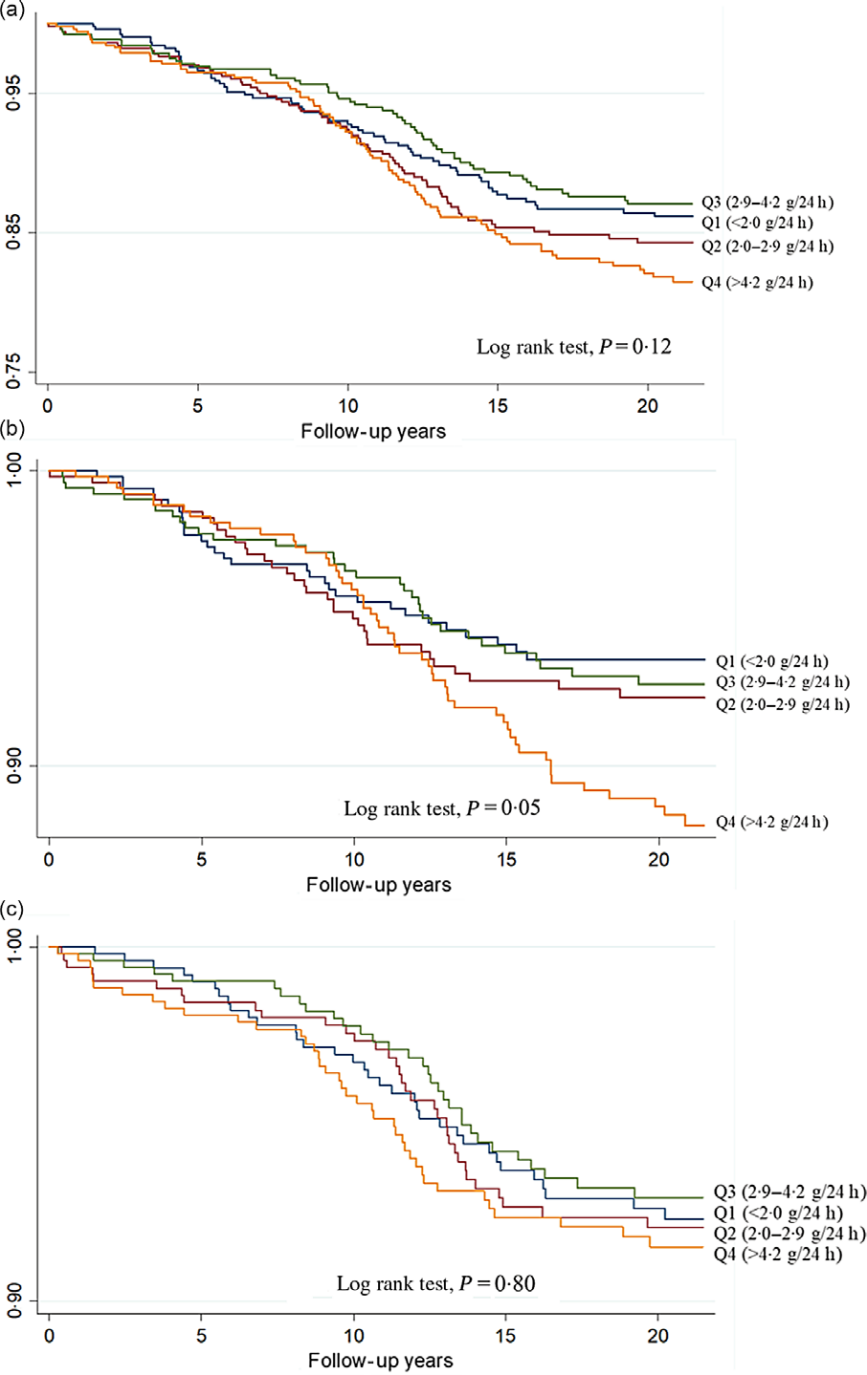
The Kaplan–Meier survival curves of CVD (a), stroke (b) and CHD according (c) to quartiles of urinary sodium excretion.

The Kaplan–Meier survival curves of CVD, CHD and stroke are presented according to quartiles of urinary Na excretion (Fig. 1). The log-rank test was not significant for CVD risk (*P* = 0·12) and CHD risk (*P* = 0·80), whereas a significant difference was observed in the log-rank test for stroke risk (*P* = 0·05). The correlations between urinary Na and other risk factors, ranging from −0·018 for LDL-cholesterol to 0·110 for SBP, are shown in Supplementary Table S1.

### Urinary sodium excretion and the risks of total CVD, CHD and stroke

During the median follow-up of 19·1 years, the total number of new cases was as follows: 279 total CVD events, 157 stroke events and 155 CHD events (Table 2). The Na excretion in the lowest quartile, as the reference values for Na, was a median of 1·5 g/24 h and mean of 1·4 g/24 h. For the risk of total CVD in model 1, compared with the first quartile with 8801·8 person-years, the HR in the highest quartile was 1.44 8814.4 person-years (95 % CI 1·04, 2·00). In model 2, the HR in the highest quartile was 1·41 *v*. the first quartile (95 % CI 1·02, 1·99). The magnitude of the total CVD risk remained significant after further adjustment for diabetes status, serum LDL-cholesterol and eGFR (adjusted HR: 1·43, 95 % CI 1·02, 1·99). The linear trend tests for the three models were significant. For the risk of CHD, compared with the first quartile, the HR in the other three quartiles were non-significant in all models. For the risk of stroke, in model 3, the result was significant (adjusted HR, 1·86; 95 % CI 1·19, 2·92). The results of the linear trend tests in the three models were all significant. A total of fifty-nine cerebral infarctions and thirty-five cerebral haemorrhage events were newly documented cases of stroke. For the risk of cerebral infarction, the HR in the highest quartile was 2·43 when adjusted for age and sex (95 % CI 1·20, 4·92). In model 2, the HR in the highest quartile was 2·63 (95 % CI 1·28, 5·38). Furthermore, the magnitude of the cerebral infarction risk remained significant after further adjustment for other potential confounders (adjusted HR, 2·50; 95 % CI 1·22, 5·15). The linear trend tests in all three models were significant. For the risk of cerebral haemorrhage, in comparison with the first quartile of urinary Na excretion, the HR in the highest quartile were non-significant in all three models. The linear trend tests in all three models were borderline significant. Linear and nonlinear associations between urinary Na excretion and the risks of total CVD, stroke and CHD were estimated. Non-significant nonlinearity was observed (online Supplementary Fig. S2, S3 and S4, respectively). Subgroup analyses were conducted and are presented in Supplementary Table S2. Men or individuals aged < 65 years with the highest urinary Na excretion had a significant HR for CVD. In addition, men, women, aged < 65 years or with eGFR of 60 ml/min per 1·73 m^2^ with the highest urinary Na excretion had a significant HR of stroke after adjusting for potential confounders.

**Table 2.** Total CVD, CHD, stroke, cerebral infarction and cerebral haemorrhage during a median of 19·1 years of follow-up according to quartiles of urinary sodium excretion* (Hazard ratios (HR) and 95% confidence intervals)

Quartiles of urinary sodium excretion	Q1	Q2		Q3		Q4		*P* for trend
		HR	95 % CI	HR	95 % CI	HR	95 % CI	
Total CVD								
Events/participants	64/528	71/528		59/528		85/528		
Rates (/1000)	7·3	8·2		6·6		9·6		
Person-year	8801·8	8676·0		8967·5		8814·4		
Model 1	1	1·11	0·79, 1·55	0·96	0·67, 1·37	1·44	1·04, 2·00	0·03
Model 2	1	1·09	0·77, 1·54	0·94	0·66, 1·34	1·41	1·02, 1·97	0·04
Model 3	1	1·03	0·73, 1·47	0·92	0·64, 1·32	1·43	1·02, 1·99	0·03
CHD								
Events/participants	33/528	36/528		31/528		57/528		
Rates (/1000)	4·0	4·3		3·6		4·3		
Person-year	8303·1	8418·0		8627·9		8556·7		
Model 1	1	1·09	0·68, 1·76	0·95	0·58, 1·55	1·20	0·75, 1·93	0·51
Model 2	1	1·11	0·69, 1·80	0·94	0·57, 1·55	1·16	0·72, 1·88	0·65
Model 3	1	1·08	0·66, 1·76	0·91	0·55, 1·51	1·16	0·72, 1·88	0·68
Stroke								
Events/participants	31/528	36/528		32/528		56/528		
Rates (/1000)	3·5	4·1		3·5		6·2		
Person-year	8832·9	8768·7		9068·4		9095·7		
Model 1	1	1·12	0·69, 1·80	1·06	0·64, 1·74	1·86	1·20, 2·89	0·002
Model 2	1	1·10	0·67, 1·79	1·03	0·63, 1·70	1·91	1·22, 2·98	0·002
Model 3	1	1·00	0·61, 1·64	1·03	0·63, 1·70	1·86	1·19, 2·92	0·001
Cerebral infarction								
Events/participants	11/528	11/528		10/528		27/528		
Model 1	1	0·93	0·40, 2·14	0·89	0·38, 2·09	2·43	1·20, 4·92	0·002
Model 2	1	0·85	0·36, 2·01	0·90	0·38, 2·14	2·63	1·28, 5·38	< 0·001
Model 3	1	0·67	0·27, 1·67	0·90	0·38, 2·14	2·50	1·22, 5·15	< 0·001
Cerebral haemorrhage								
Events/participants	5/528	7/528		11/528		12/528		
Model 1	1	1·37	0·43, 4·33	2·26	0·78, 6·53	2·48	0·87, 7·09	0·06
Model 2	1	1·38	0·43, 4·37	2·37	0·81, 4·37	2·51	0·87, 7·20	0·06
Model 3	1	1·34	0·42, 4·26	2·52	0·86, 7·36	2·58	0·89, 7·44	0·05

* Model 1: Adjusted for age groups (35–44, 45–54, 55–64, 65–74 and ≥75 years of age) and sex. Model 2: Model 1 plus BMI (<18, 18–20·9, 21–22·9, 23–24·9 or ≥25 kg/m^2^), smoking (yes/no or abstinence), current alcohol drinking (regular/no), marital status (single, married and living with a spouse, or divorced and separated), regular exercise habits (yes/no), education level (<9 years, at least 9 years) and occupation (no work, labour, official or business). Model 3: Model 2 plus LDL (mg/dl), glomerular filtration rate (<60, ≥60 ml/min per 1·73 m^2^) and diabetes status (yes/no).

The analyses of the effect of hypertension on the relationship between urinary Na excretion and the risks of total CVD, CHD and stroke are shown in Table 3. For the risk of total CVD, the HR in normotensive participants with high urinary Na excretion was 1·43 (95 % CI 0·94, 2·16), the HR in hypertensive participants with low urinary Na excretion was 2·71 (95 % CI 1·63, 4·50) and the HR in hypertensive participants with high urinary Na excretion was 2·64 (95 % CI 1·71, 4·07) in model 3. For the risk of CHD, the HR in normotensive participants with high urinary Na excretion was 1·64 (95 % CI 0·92, 2·95), which was non-significant in model 3. In contrast, the significant HR in hypertensive participants with low urinary Na excretion was 2·88 (95 % CI 1·41, 3·91) and the HR in hypertensive participants with high urinary Na excretion was 2·08 (95 % CI 1·11, 3·91) in model 3. For the risk of stroke, a non-significant HR in normotensive participants with high urinary Na excretion (adjusted HR: 1·31, 95 % CI 0·74, 2·32). Conversely, the significant HR in hypertensive participants with low urinary Na excretion was 2·20 (95 % CI 1·08, 4·49), and the HR in hypertensive participants with high urinary Na excretion was 2·95 (95 % CI 1·65, 5·28) in model 3. The interaction effect between hypertension and Na excretion was significant for CHD risk (*P* = 0·04). The effect of the metabolic syndrome status on the association between urinary Na excretion and the risks of total CVD, CHD and stroke are presented in Supplementary Table S3. The participants with the metabolic syndrome status had significant HR for total CVD, CHD and stroke in the three models. The effect of overweight status on Na–CVD association is shown in Supplementary Table S4. Only overweight participants with high Na excretion had significant HR for CHD in the three models. The effects of potential confounders and 24-h potassium excretion were similar to those of the models without adjusting for 24-h potassium excretion (online Supplementary Table S5 and S6).

**Table 3.** Total CVD, CHD and stroke according to the urinary sodium excretion and hypertension* (Hazard ratios (HR) and 95% confidence intervals)

	Normotensive			Hypertensive			
Urinary Na excretion (g/24 h)	Low (<2 g/d)	High (≥2 g/d)		Low (<2 g/d)		High (≥2 g/d)	
		HR	95 % CI	HR	95 % CI	HR	95 % CI
Total CVD							
Events/participants	29/438	130/1193		34/121		83/354	
Model 1	1	1·41	0·94, 2·13	2·78	1·69, 4·56	2·88	1·89, 4·38
Model 2	1	1·39	0·92, 2·09	2·72	1·65, 4·47	2·76	1·81, 4·22
Model 3	1	1·43	0·94, 2·16	2·71	1·63, 4·50	2·64	1·71, 4·07
CHD							
Events/participants	18/438	72/1193		17/121		34/354	
Model 1	1	1·63	0·92, 2·92	3·13	1·55, 4·70	2·54	1·37, 4·70
Model 2	1	1·64	0·92, 2·94	3·02	1·49, 6·11	2·35	1·27, 4·37
Model 3	1	1·64	0·92, 2·95	2·88	1·41, 3·91	2·08	1·11, 3·91
Stroke							
Events/participants	15/438	68/1193		17/121		53/354	
Model 1	1	1·28	0·73, 2·23	2·12	1·06, 4·24	2·89	1·66, 2·48
Model 2	1	1·27	0·73, 2·21	2·12	1·68, 5·17	2·95	1·68, 5·17
Model 3	1	1·31	0·74, 2·32	2·20	1·08, 4·49	2·95	1·65, 5·28

* Model 1: Adjusted for age groups (35–44, 45–54, 55–64, 65–74 ≥75 years of age) and sex. Model 2: Model 1 plus BMI (<18, 18–20·9, 21–22·9, 23–24·9 or ≥25 kg/m^2^), smoking (yes/no or abstinence), current alcohol drinking (regular/no), marital status (single, married and living with a spouse, or divorced and separated), regular exercise habits (yes/no), education level (<9 years, at least 9 years) and occupation (no work, labour, official or business). Model 3: Model 2 plus LDL (mg/dl), glomerular filtration rate (<60, ≥60 ml/min per 1·73 m^2^) and diabetes status (yes/no). *P* for interaction for total CVD: 0·19. *P* for interaction for CHD: 0·04. *P* for interaction for stroke: 0·94.

### The mediation effects on the sodium–CVD association

**Fig. 2.**
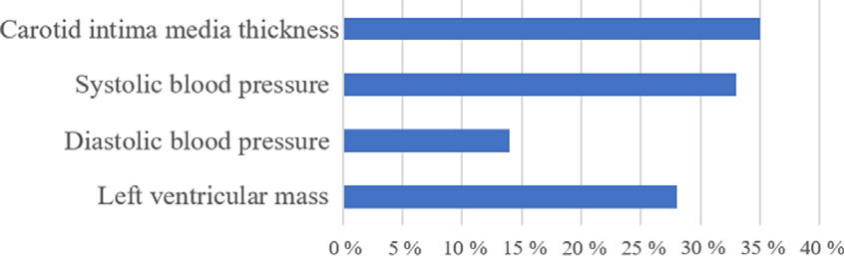
The proportion of CVD risk reduction for the highest group of urinary sodium excretion in the traditional mediation approach. 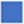, Reduction.

In the traditional mediation approach, the proportion of urinary Na excretion increasing in the CVD risk explained by each potential mediator was calculated and is shown in Fig. 2. The carotid intima-media thickness had the largest contribution to urinary Na excretion–CVD risk (35 %), followed by SBP (33 %), left ventricular mass (28 %) and DBP (14 %). All four mediating effects were significant in the traditional mediation approach (Supplementary Table S7). A sensitivity analysis was performed using the counterfactual framework approach. Similar mediation effects were observed using both the traditional and counterfactual framework approaches (Supplementary Fig. S5).

## Discussion

### Main findings

In this study, the participants with the highest urinary Na excretion had a significant risk of total CVD, stroke and cerebral infarction. In addition, hypertensive individuals with urinary Na excretion <2 g have the highest risks of total CVD, CHD and stroke. Participants with the metabolic syndrome and urinary Na excretion >2 g had the highest risk of CVD, CHD and stroke. In addition, this current study indicates that the relationship between higher urinary Na excretion and the risk of CVD can be attributed to carotid intima-media thickness, SBP, left ventricular mass and DBP, in descending order according to the traditional mediation approach.

### Comparison with previous studies

Our results are consistent with prior evidence, which indicates that the highest urinary Na excretion was associated with significant risks of CVD and stroke^(52)^. In addition, a significant linear relationship between dietary Na intake and risk of stroke was found in our study. The linear association was in accordance with the results of previous studies^(53)^. However, a few studies demonstrated that there was no association between dietary Na intake and the risk of stroke^(54,55)^. Such differences between the studies may be due to the variance of assessment of dietary Na and the purpose of the different studies. Individuals living in different countries have different dietary patterns of Na intake for many reasons, such as various cultures and food availability. Furthermore, an individual’s diet could change from day to day or from workday to weekend^(56,57)^. In addition, the purpose of these studies may lead to different results. Compared with the studies assessing an individual’s Na intake, the studies designed to evaluate the average Na consumption of a population had little random error. The random error may not influence the results of the population-based studies but could lead to inaccurate estimates of the results of the individual-based studies^(58)^.

Our analysis indicated that individuals with hypertension or the metabolic syndrome had significant risks of CVD, CHD and stroke regardless of high or low urinary Na excretion. Previous studies showed that higher urinary Na excretion was associated with risks of hypertension, the metabolic syndrome and CVD^(10,59–61)^. Moreover, hypertension and the metabolic syndrome are widely thought of as two independent risk factors of CVD^(62)^. Many factors result in hypertension and the metabolic syndrome, including high urinary Na excretion; once an individual experiences hypertension or the metabolic syndrome, which by themselves are CVD risk factors, the effect of Na intake is weakened on CVD. As a result, we conducted a mediation analysis to test whether hypertension and the metabolic syndrome were mediators. However, the metabolic syndrome did not meet the criteria in the Baron and Kenny approach, making it unsuitable for causal inference as a mediator between high urinary Na excretion and CVD risk. Previous studies showed that a high Na intake was associated with the metabolic syndrome^(61)^. Furthermore, a study in 2012 with a total of 716 middle-aged participants indicated that higher dietary Na intake was an independent dietary indicator of the metabolic syndrome^(63)^. Therefore, although the analysis results showed that even if urinary Na excretion was high, people without hypertension or the metabolic syndrome were not at high risk for CVD, it is still necessary to modify lifestyle factors related to hypertension and the metabolic syndrome including Na reduction to reduce the risk of CVD. The causal relationship between dietary Na intake and the metabolic syndrome should be further explored in experimental studies or randomised clinical trials. In addition, since we did not give participants high-salt and low-salt diet at baseline, we cannot infer that participants with hypertension or the metabolic syndrome were salt-sensitive. More studies in which researchers separated persons into salt-sensitive or salt-resistance at baseline are necessary in the future in Taiwan.

### Potential biological mechanisms

In the mediation analysis, our results are consistent with previous studies showing that SBP, DBP, carotid intima-media thickness and left ventricular mass are potential mediators in the relationship between high urinary Na excretion and the risk of CVD. First, high Na intake may trigger water retention in the body and then provoke high flow in arterial vessels, which activates the pressure natriuresis mechanism^(64)^. Subsequently, the renal arteries with raised BP induce increased Na and water excretion. Some animal studies have indicated that such hemodynamic load may cause adverse microvascular remodelling due to high-Na-intake-induced increased BP and vascular resistance^(65–67)^. Moreover, a higher BP is widely recognised as a risk factor for CVD^(68)^. Many clinical trials have also provided evidence that a direct causal relationship exists between Na intake and increased BP^(69,70)^. Second, excessive Na intake has been shown to cause atherosclerosis through raising carotid intima-media thickness, independent from increasing BP. Ferreira-Sae and colleagues examined the association between dietary Na intake and the changes in carotid artery structure among a population of 134 hypertensive participants. The study indicated that dietary Na intake was significantly associated with carotid intima-media thickness (*r* = 0·19, *P* < 0·05) and internal carotid artery resistive index (*r* = 0·20; *P* < 0·05) in the univariate analysis. This study implies that excess Na intake may cause alterations in the carotid structure^(71)^. In addition, a clinical trial of 258 normotensive and obese participants found a significant positive association between increased carotid intima-media thickness and higher urinary Na excretion quartile^(72)^. Ustundag *et al.* performed a study with 193 patients with chronic kidney disease and found a relationship between urinary Na excretion and carotid intima-media thickness, which implied that excessive Na intake potentially led to the development of atherosclerosis in patients with chronic kidney disease^(73)^. In addition to the carotid intima-media thickness effect, high urinary Na excretion has been found to lead to increased left ventricular mass. Rodriguez *et al.* conducted a cohort study of adults aged 18–30 years^(74)^. Five years later, the risk of left ventricular mass related to urinary Na excretion was 2·65 (95 % CI 1·65, 4·26) after adjustment for SBP. In 1041 participants, for each standard deviation unit increment of Na excretion, the left ventricular mass averaged 0·88 g/m^2·7^ higher (*P* = 0·01)^(74)^. The four biomarkers, SBP, DBP, carotid intima-media thickness and left ventricular mass, are reported to be risk factors for the development of CVD. Our study provided evidence that the relationship between high urinary Na excretion and the risk of CVD was potentially mediated by elevated SBP and DBP, increased carotid intima-media thickness and increased left ventricular mass.

### Clinical implications

Our results indicated that high urinary Na excretion was associated with a higher risk of CVD, particularly stroke. In addition, SBP, DBP, carotid intima-media thickness and left ventricular mass were the potential mediators linking urinary Na excretion and CVD risk. Individuals without CVD should be encouraged to use Na reduction strategies to prevent the development of CVD. Individuals with hypertension or the metabolic syndrome should not only reduce Na intake but also actively control the factors in the metabolic syndrome to normal conditions for primary prevention.

### Strengths and limitations

The strengths of the current study included three aspects: the prospective epidemiological design with longitudinal follow-up, the focus of primary prevention and the mediated effect linking urinary Na excretion and CVD risk. The follow-up time in the current cohort data was from 1992 to 2013, which may be long enough to observe the incidence of CVD. To the best of our knowledge, this study is the first to investigate the association between urinary Na excretion and the risk of CVD, CHD and stroke in Taiwan. In addition, as we excluded individuals with CVD events at baseline, all the participants enrolled were free of any type of CVD. Hence, the results affirmed that different levels of urinary Na excretion, representing dietary Na intake, had different risks of CVD, especially the risk of stroke. Finally, the current study is the first to explore possible mediators between Na excretion and CVD risk. We used two mediation analysis approaches to examine the mediators linking high Na excretion and increased CVD risk. Because clinical trials for dietary Na intake and CVD risk are difficult to implement, mediation analysis in epidemiology can provide evidence to illustrate the underlying mechanisms.

Despite the appropriate epidemiological design, there were some limitations to this study. First, we did not collect detailed medication use by the participants. The urinary Na excretion of individuals with kidney dysfunction may not represent their usual Na intake. In fact, patients taking diuretics may represent a bias when their urinary Na excretion is regarded as usual Na intake. However, we conducted a subgroup analysis stratified by eGFR of ≥60 ml/min per 1·73 m^2^ or not. The results proved that there was no difference between the two groups in the risk of CVD and CHD. Second, we collected only a single overnight urine sample. Some other formulas which can calculate 24-h urine Na excretion using a spot or overnight urine were shown in previous studies^(19,75–77)^. The bias of overnight urine collection has been reported as a non-differential misclassification, which may minimise the effect. In previous studies, multiple 24-h urinary Na collections have been validated to ensure reliability^(78,79)^. However, the disadvantages of 24-h urine collection may be low response rates and high burden in population surveys. Hence, accessibility and affordability are the irreplaceable characteristics of overnight urine collection in public health. In addition, the participants were reminded not to change their dietary habits during the collection period so that the urine samples were appropriate to represent their usual Na intake. Moreover, we used the Tanaka formula^(19)^, which took into account urine creatinine excretion to correct the 24-h urine Na excretion to test the correlation with our data. The correlation coefficient between the estimated 24-h urine excretion calculated by the Kawasaki formula and the Tanaka formula is 0·47 (*P* < 0·001) (online Supplementary Fig. S6). As a result, overnight urine samples in our study were a practical and affordable substitute for 24-h urine excretion. Third, external validity was limited because the participants in the CCCC data were only from one community. National research on the relationship between Taiwanese dietary Na intake and the risk of CVD in the future is necessary. In summary, we believe that the current study provides cogent evidence despite the existing non-differential misclassification. Fourth, as we did not collect the dietary habits of each participant, we could not determine what food the participants consumed that caused the Na intake to increase. Although differences in dietary patterns may affect the risk of CVD, Na intake obtained from dietary records or dietary frequency questionnaires is usually inaccurate. In contrast, dietary Na intake estimated from urine collection is more objective and can reduce self-reporting bias. Further studies to explore the effect of consumption frequency of foods high in Na on the prevention of CVD are necessary.

### Conclusions

The findings of this study suggested that high urinary Na excretion was associated with a higher risk of CVD, especially stroke. The results implied that individuals with hypertension and the metabolic syndrome were more sensitive to excessive Na intake and had a higher risk of CVD. Na restriction and control of the metabolic syndrome and hypertension are encouraged. Further studies on salt sensitivity are warranted.

## Supplementary material

For supplementary material accompanying this paper visit https://doi.org/10.1017/S0007114521001768. click here to view supplementary material
