# Supplementary material for: Urinary sodium excretion and the risk of CVD: a community-based cohort study in Taiwan
Source: Br J Nutr. 2021 May 27;127(7):1086–97. doi: 10.1017/S0007114521001768 (PMC8924491; doi:10.1017/S0007114521001768)
Supplement: Supplementary file 1 [file S0007114521001768sup001.docx]

**Supplementary data**

**YJ Wang, et al. Urinary sodium excretion and the risk of cardiovascular disease: a community-based cohort study in Taiwan**

**Table S1.** Unadjusted Spearman partial correlation coefficients between various measurements and urinary sodium excretion among the study participants in the CCCC Study.

**Table S2.** Subgroup analyses for the risks of cardiovascular disease, CHD, and stroke according to the urinary sodium excretion categories

**Table S3.** Hazard ratios and 95% confidence intervals of total cardiovascular disease, coronary heart disease, and stroke according to the urinary sodium excretion and the metabolic syndrome status

**Table S4.** Hazard ratios and 95% confidence intervals of total cardiovascular disease, coronary heart disease, and stroke according to the urinary sodium excretion and the overweight status

**Table S5.** Hazard ratios and 95% confidence intervals of total cardiovascular disease, coronary heart disease, stroke, cerebral infarction, and cerebral hemorrhage according to quartiles of urinary sodium excretion after adjusted for potential confounders and 24-hour potassium excretion.

**Table S6.** Hazard ratios and 95% confidence intervals of total cardiovascular disease, coronary heart disease, and stroke according to the urinary sodium excretion and hypertension after adjusted for potential confounders and 24-hour potassium excretion.

**Table S7.** Association of urinary sodium excretion with incident CVD after adjustment for potential mediators.

**Figure S1**. Study flow chart of this study

**Figure S2.** The linear (A) and non-linear (B) association of urinary sodium excretion and HR of cardiovascular disease.

**Figure S3**. The linear (A) and non-linear (B) association of urinary sodium excretion and HR of stroke.

**Figure S4**. The linear (A) and non-linear (B) association of urinary sodium excretion and HR of coronary heart disease.

**Figure S5**. The proportion of CVD risk reduction for the highest group of urinary sodium excretion in the counterfactual framework approach.

**Figure S6.** Scatterplots and correlations for overnight urine sodium excretion, estimated 24-hour urine sodium excretion by the Kawasaki formula, and estimated 24-hour urine sodium excretion by the Tanaka formula.

**Table S1. Unadjusted Spearman partial correlation coefficients between various measurements and urinary sodium excretion among the study participants in the CCCC Study.**

|  | SBP | DBP | CIMT | LVM | BMI | Metabolic syndrome | Uric acid | LDL-C | HDL-C | Fasting glucose |
| --- | --- | --- | --- | --- | --- | --- | --- | --- | --- | --- |
| Urinary sodium | 0.110*** | 0.082*** | 0.048* | 0.060*** | 0.136*** | 0.032 | -0.037 | -0.018 | 0.054 | 0.068** |

Abbreviations: BMI, body mass index; CIMT, Carotid Intima-Media Thickness; DBP, diastolic blood pressure; HDL-C, high-density lipoprotein cholesterol; LDL-C, low-density lipoprotein cholesterol; LVM, Left ventricular mass; SBP, systolic blood pressure.

* P<0.05. ** P<0.01. *** P<0.001.

## Table S2. Subgroup analyses for the risks of cardiovascular disease, CHD, and stroke according to the urinary sodium excretion categories.

|  | Quartiles of urinary sodium excretion, HR (95% CI) | | | | | P for interaction |
| --- | --- | --- | --- | --- | --- | --- |
|  | Q1 | Q2 | Q3 | Q4 | P for trend |  |
| Cardiovascular disease | | | | | | |
| Gender^a^ |  |  |  |  |  | 0.81 |
| Men (n/N) | 36/255 | 46/246 | 29/241 | 49/233 |  |  |
| HR | 1 | 1.28 (0.81, 2.03) | 0.88 (0.53, 1.46) | 1.69 (1.08, 2.64) | 0.04 |  |
| Women (n/N) | 28/273 | 25/282 | 30/287 | 36/295 |  |  |
| HR | 1 | 0.84 (0.49, 1.46) | 1.02 (0.60, 1.71) | 1.22 (0.74, 2.02) | 0.27 |  |
| Age, yrs^b^ |  |  |  |  |  | 0.21 |
| <65 (n/N) | 38/412 | 40/406 | 33/433 | 61/440 |  |  |
| HR | 1 | 1.03 (0.65, 1.62) | 0.79 (0.49, 1.26) | 1.51 (1.00, 2.28) | 0.03 |  |
| ≥65 (n/N) | 26/116 | 31/122 | 26/95 | 24/88 |  |  |
| HR | 1 | 1.04 (0.61, 1.78) | 1.09 (0.62, 1.91) | 1.24 (0.71, 2.19) | 0.43 |  |
| Menopause status ^d^ |  |  |  |  |  | 0.77 |
| Non-menopause (n/N) | 1/100 | 5/118 | 5/105 | 8/114 |  |  |
| HR | 1 | 2.68 (0.29, 24.64) | 4.42 (0.50, 39.28) | 7.52 (0.92, 61.85) | 0.02 |  |
| Menopause (n/N) | 24/153 | 19/142 | 19/160 | 25/160 |  |  |
| HR | 1 | 0.87 (0.47, 1.60) | 0.76 (0.41, 1.40) | 1.01 (0.57, 1.79) | 0.92 |  |
| eGFR, mL/min/1.73 m^2 c^ |  |  |  |  |  | 0.96 |
| ≥60 (n/N) | 47/397 | 56/415 | 46/420 | 67/415 |  |  |
| HR | 1 | 1.06 (0.71, 1.57) | 0.90 (0.60 1.36) | 1.43 (0.88, 2.23) | 0.06 |  |
| <60 (n/N) | 17/127 | 14/111 | 12/105 | 17/110 |  |  |
| HR | 1 | 0.80 (0.37, 1.72) | 1.00 (0.46, 2.17) | 1.28 (0.63, 2.66) | 0.35 |  |
| Coronary heart disease | | | | | | |
| Gender^a^ |  |  |  |  |  | 0.44 |
| Men (n/N) | 19/241 | 25/237 | 17/237 | 25/230 |  |  |
| HR | 1 | 1.36 (0.73, 2.54) | 0.90 (0.46, 1.77) | 1.51 (0.81, 2.83) | 0.32 |  |
| Women (n/N) | 14/250 | 11/269 | 14/264 | 12/275 |  |  |
| HR | 1 | 0.69 (0.31, 1.52) | 0.87 (0.41, 1.86) | 0.70 (0.32, 1.55) | 0.53 |  |
| Age, yrs^b^ |  |  |  |  |  | 0.49 |
| <65 (n/N) | 21/385 | 21/387 | 19/411 | 27/420 |  |  |
| HR | 1 | 0.99 (0.53, 1.83) | 0.77 (0.41, 1.46) | 1.10 (0.61, 1.97) | 0.76 |  |
| ≥65 (n/N) | 12/106 | 15/119 | 12/90 | 10/85 |  |  |
| HR | 1 | 0.98 (0.45, 2.12) | 0.94 (0.41, 2.17) | 0.97 (0.40, 2.34) | 0.93 |  |
| eGFR, mL/min/1.73 m^2 c^ |  |  |  |  |  | 0.41 |
| ≥60 (n/N) | 24/365 | 30/392 | 22/396 | 27/394 |  |  |
| HR | 1 | 1.16 (0.67, 2.00) | 0.82 (0.45, 1.48) | 1.09 (0.62, 1.90) | 0.99 |  |
| <60 (n/N) | 9/122 | 6/112 | 8/102 | 9/108 |  |  |
| HR | 1 | 0.59 (0.18, 1.90) | 1.17 (0.44, 3.14) | 1.34 (0.49, 3.69) | 0.32 |  |
| Menopause status ^d^ |  |  |  |  |  |  |
| Non-menopause (n/N) | 1/92 | 3/112 | 4/94 | 2/108 |  | 0.28 |
| HR | 1 | 1.72 (0.16, 18.46) | 2.55 (0.26, 25.38) | 1.58 (0.13, 19.12) | 0.76 |  |
| Menopause (n/N) | 12/140 | 8/136 | 8/150 | 10/147 |  |  |
| HR | 1 | 0.60 (0.24, 1.50) | 0.54 (0.21, 1.39) | 0.68 (0.28, 1.61) | 0.48 |  |
| Stroke | | | | | | |
| Gender^a^ |  |  |  |  |  | 0.78 |
| Men (n/N) | 18/251 | 22/246 | 16/242 | 31/235 |  |  |
| HR | 1 | 1.10 (0.56, 2.13) | 0.96 (0.48, 1.93) | 1.96 (1.07, 3.61) | 0.02 |  |
| Women (n/N) | 13/271 | 14/283 | 16/284 | 25/294 |  |  |
| HR | 1 | 1.06 (0.49, 2.29) | 1.23 (0.59, 2.56) | 2.01 (1.02, 3.96) | 0.02 |  |
| Age, yrs^b^ |  |  |  |  |  | 0.16 |
| <65 (n/N) | 15/405 | 20/409 | 16/431 | 39/438 |  |  |
| HR | 1 | 1.19 (0.59, 2.37) | 1.05 (0.52, 2.15) | 2.58 (1.41, 4.73) | <0.001 |  |
| ≥65 (n/N) | 16/117 | 16/120 | 16/95 | 17/91 |  |  |
| HR | 1 | 0.92 (0.45, 1.88) | 1.14 (0.56, 2.32) | 1.31 (0.65, 2.66) | 0.35 |  |
| eGFR, mL/min/1.73 m^2 c^ |  |  |  |  |  | 0.76 |
| ≥60 (n/N) | 23/393 | 26/414 | 25/419 | 45/416 |  |  |
| HR | 1 | 0.99 (0.56, 1.76) | 1.00 (0.56, 1.77) | 1.92 (1.16, 3.19) | 0.003 |  |
| <60 (n/N) | 8/125 | 8/112 | 7/104 | 10/110 |  |  |
| HR | 1 | 0.97 (0.34, 2.79) | 1.25 (0.43, 3.59) | 1.60 (0.59, 4.34) | 0.28 |  |
| Menopause status ^d^ |  |  |  |  |  | 0.02 |
| Non-menopause (n/N) | 0/97 | 2/118 | 2/103 | 6/114 |  |  |
| HR | 1 | NA ^e^ | NA ^e^ | NA ^e^ | 0.01 |  |
| Menopause (n/N) | 11/153 | 11/143 | 11/160 | 16/159 |  |  |
| HR | 1 | 0.87 (0.39, 2.21) | 0.89 (0.38, 2.08) | 1.42 (0.65, 3.11) | 0.25 |  |

Abbreviations: eGFR, estimation of glomerular filtration rate; HR, hazard ratio

a: Model was adjusted for age (35–44, 45–54, 55–64, 65–74, ≥75 years of age), body mass index (<18, 18 to 20.9, 21 to 22.9, 23 to 24.9, or ≥25 kg/m^2^), smoking (yes/no or abstinence), current alcohol drinking (regular/no), marital status (single, married and living with a spouse, or divorced and separated), regular exercise habit (yes/no), an education level (less than 9 years, at least 9 years), occupation (no work, labor, official or business), low-density lipoprotein (mg/dL), and glomerular filtration rate (<60,>=60 mL/min/1.73 m^2^).

b: Model was adjusted for sex, body mass index (<18, 18 to 20.9, 21 to 22.9, 23 to 24.9, or ≥25 kg/m^2^), smoking (yes/no or abstinence), current alcohol drinking (regular/no), marital status (single, married and living with a spouse, or divorced and separated), regular exercise habit (yes/no), an education level (less than 9 years, at least 9 years), occupation (no work, labor, official or business), low-density lipoprotein (mg/dL), and glomerular filtration rate (<60,>=60 mL/min/1.73 m^2^).

c:Model was adjusted for age (35–44, 45–54, 55–64, 65–74, ≥75 years of age), sex, body mass index (<18, 18 to 20.9, 21 to 22.9, 23 to 24.9, or ≥25 kg/m^2^), smoking (yes/no or abstinence), current alcohol drinking (regular/no), marital status (single, married and living with a spouse, or divorced and separated), regular exercise habit (yes/no), an education level (less than 9 years, at least 9 years), occupation (no work, labor, official or business), and low-density lipoprotein (mg/dL).

d: Model was adjusted for age (35–44, 45–54, 55–64, 65–74, ≥75 years of age), sex, body mass index (<18, 18 to 20.9, 21 to 22.9, 23 to 24.9, or ≥25 kg/m^2^), smoking (yes/no or abstinence), current alcohol drinking (regular/no), marital status (single, married and living with a spouse, or divorced and separated), regular exercise habit (yes/no), an education level (less than 9 years, at least 9 years), occupation (no work, labor, official or business), low-density lipoprotein (mg/dL), and glomerular filtration rate (<60,>=60 mL/min/1.73 m^2^).

e: Estimated hazard ratios cannot be calculated because none of the events occurred in the reference group.

## Table S3. Hazard ratios and 95% confidence intervals of total cardiovascular disease, coronary heart disease, and stroke according to the urinary sodium excretion and the metabolic syndrome status.

|  | Non-metabolic syndrome | | Metabolic syndrome | |
| --- | --- | --- | --- | --- |
| Urinary sodium excretion (g/24hr) | Low (<2g/day) | High (>=2g/day) | Low (<2g/day) | High (>=2g/day) |
| Total cardiovascular disease | | | | |
| Events/participants | 31/396 | 121/1096 | 35/165 | 92/455 |
| Model 1 | 1 | 1.42 (0.96, 2.11) | 2.71 (1.66, 4.41) | 2.94 (1.94, 4.44) |
| Model 2 | 1 | 1.38 (0.93, 2.06) | 2.62 (1.59, 4.33) | 2.84 (1.84, 4.37) |
| Model 3 | 1 | 1.36 (0.91, 2.02) | 2.53 (1.53, 4.19) | 2.67 (1.73, 4.13) |
| Coronary heart disease | | | | |
| Events/participants | 16/396 | 57/1096 | 19/165 | 49/455 |
| Model 1 | 1 | 1.30 (0.73, 2.30) | 3.03 (1.52, 6.05) | 3.39 (1.89, 6.08) |
| Model 2 | 1 | 1.26 (0.71, 2.25) | 2.49 (1.22, 5.08) | 2.85 (1.55, 5.24) |
| Model 3 | 1 | 1.22 (0.68, 2.17) | 2.45 (1.20, 5.00) | 2.70 (1.46, 5.00) |
| Stroke | | | | |
| Events/participants | 16/396 | 71/1096 | 17/165 | 50/455 |
| Model 1 | 1 | 1.59 (0.93, 2.74) | 2.32 (1.16, 4.63) | 2.62 (1.48, 4.65) |
| Model 2 | 1 | 1.60 (0.93, 2.75) | 2.62 (1.30, 5.30) | 2.94 (1.63, 5.33) |
| Model 3 | 1 | 1.55 (0.90, 2.68) | 2.52 (1.24, 5.15) | 2.91 (1.60, 5.29) |

Model 1: adjusted for age (35–44, 45–54, 55–64, 65–74, ≥75 years of age) and sex

Model 2: Model 1 plus body mass index (<18, 18 to 20.9, 21 to 22.9, 23 to 24.9, or ≥25 kg/m^2^), smoking (yes/no or abstinence), current alcohol drinking (regular/no), marital status (single, married and living with a spouse, or divorced and separated), regular exercise habit (yes/no), an education level (less than 9 years, at least 9 years), occupation (no work, labor, official or business)

Model 3: Model 2 plus low-density lipoprotein (mg/dL), and glomerular filtration rate (<60,>=60 mL/min/1.73 m^2^)

P for interaction for total cardiovascular disease: 0.39

P for interaction for coronary heart disease: 0.81

P for interaction for stroke: 0.46

## Table S4. Hazard ratios and 95% confidence intervals of total cardiovascular disease, coronary heart disease, and stroke according to the urinary sodium excretion and the overweight status.

|  | Non-overweight (BMI<23.0) | | Overweight (BMI>=23.0) | |
| --- | --- | --- | --- | --- |
| Urinary sodium excretion (g/24hr) | Low (<2g/day) | High (>=2g/day) | Low (<2g/day) | High (>=2g/day) |
| Total cardiovascular disease | | | | |
| Events/participants | 32/288 | 82/694 | 34/270 | 130/855 |
| Model 1 | 1 | 1.00 (0.66, 1.50) | 1.04 (0.64, 1.69) | 1.43(0.97, 2.12) |
| Model 2 | 1 | 0.98 (0.65, 1.48) | 1.04 (0.64, 1.69) | 1.45 (0.98, 2.14) |
| Model 3 | 1 | 1.00 (0.66, 1.51) | 0.98 (0.60, 1.59) | 1.32 (0.89, 1.96) |
| Coronary heart disease | | | | |
| Events/participants | 14/288 | 32/694 | 21/270 | 73/855 |
| Model 1 | 1 | 0.88(0.46, 1.69) | 1.49 (0.74, 3.00) | 1.98 (1.09, 3.59) |
| Model 2 | 1 | 0.88 (0.46, 1.68) | 1.46 (0.72, 2.95) | 2.03 (1.12, 3.69) |
| Model 3 | 1 | 0.91 (0.47, 1.75) | 1.44 (0.71, 2.91) | 1.88 (1.03, 3.44) |
| Stroke | | | | |
| Events/participants | 18/288 | 53/694 | 15/270 | 68/855 |
| Model 1 | 1 | 1.18 (0.70, 2.01) | 0.79 (0.40, 1.58) | 1.20 (0.71, 2.02) |
| Model 2 | 1 | 1.16 (0.68, 1.98) | 0.79 (0.40, 1.57) | 1.21 (0.71, 2.04) |
| Model 3 | 1 | 1.15 (0.67, 1.97) | 0.72 (0.36, 1.45) | 1.11 (0.65, 1.89) |

Model 1: adjusted for age (35–44, 45–54, 55–64, 65–74, ≥75 years of age) and sex

Model 2: Model 1 plus smoking (yes/no or abstinence), current alcohol drinking (regular/no), marital status (single, married and living with a spouse, or divorced and separated), regular exercise habit (yes/no), an education level (less than 9 years, at least 9 years), occupation (no work, labor, official or business)

Model 3: Model 2 plus low-density lipoprotein (mg/dL), glomerular filtration rate (<60,>=60 mL/min/1.73 m^2^), and diabetes status (yes/no)

P for interaction for total cardiovascular disease:0.29

P for interaction for coronary heart disease:0.40

P for interaction for stroke:0.47

**Table S5. Hazard ratios and 95% confidence intervals of total cardiovascular disease, coronary heart disease, stroke, cerebral infarction, and cerebral hemorrhage according to quartiles of urinary sodium excretion after adjusted for potential confounders and 24-hour potassium excretion.**

| Quartiles of urinary sodium excretion | Q1 | Q2 | Q3 | Q4 | P for trend |
| --- | --- | --- | --- | --- | --- |
| Total cardiovascular disease | 1 | 1.07 (0.75, 1.51) | 0.97 (0.66, 1.40) | 1.60 (1.10, 2.31) | 0.01 |
| Coronary heart disease | 1 | 1.13 (0.68, 1.87) | 1.00 (0.59, 1.69) | 1.34 (0.78, 2.32) | 0.34 |
| Stroke | 1 | 0.98 (0.59, 1.61) | 0.98 (0.58, 1.64) | 1.75 (1.06, 2.86) | 0.009 |
| Cerebral infarction | 1 | 0.64 (0.25, 1.61) | 0.81 (0.33, 1.98) | 2.16 (0.97, 4.84) | 0.007 |
| Cerebral hemorrhage | 1 | 1.25 (0.39, 4.03) | 2.20 (0.72, 6.69) | 2.37 (0.76, 7.42) | 0.09 |

Adding adjusted for 24-hour potassium excretion groups (<17.4, 17.4 to 24.3, 24.3 to 33.0, ≥28.3 mmol)

**Table S6. Hazard ratios and 95% confidence intervals of total cardiovascular disease, coronary heart disease, and stroke according to the urinary sodium excretion and hypertension** **after adjusted for potential confounders and 24-hour potassium excretion.**

|  | | Normotensive | | | Hypertensive | |
| --- | --- | --- | --- | --- | --- | --- |
| Urinary sodium excretion (g/24hr) | Low (<2g/day) | | High (>=2g/day) | Low (<2g/day) | | High (>=2g/day) |
| Total cardiovascular disease | 1 | | 1.46 (0.96, 2.24) | 2.69 (1.62, 4.47) | | 2.72 (1.74, 4.24) |
| Coronary heart disease | 1 | | 1.80 (0.99 ,3.27) | 2.83 (1.39, 5.78) | | 2.32 (1.21, 4.42) |
| Stroke | 1 | | 1.20 (0.67, 2.15) | 2.23 (1.09, 4.56) | | 2.67 (1.48, 4.84) |
|  | | Non-metabolic syndrome | | | Metabolic syndrome | |
| Total cardiovascular disease | 1 | | 1.39 (0.92, 2.08) | 2.52 (1.52, 4.18) | | 2.74 (1.75, 4.28) |
| Coronary heart disease | 1 | | 1.32 (0.73, 2.39) | 2.40 (1.17, 4.91) | | 2.99 (1.59, 5.62) |
| Stroke | 1 | | 1.41 (0.81, 2.47) | 2.62 (1.28, 5.35) | | 2.58 (1.40, 4.75) |
|  | | Non-overweight (BMI<23.0) | | | Overweight (BMI>=23.0) | |
| Total cardiovascular disease | 1 | | 1.03 (0.67, 1.57) | 0.98 (0.60, 1.61) | | 1.37 (0.91, 2.07) |
| Coronary heart disease | 1 | | 0.98 (0.50, 1.91) | 1.44 (0.71, 2.93) | | 2.12 (1.13, 3.96) |
| Stroke | 1 | | 1.06 (0.61, 1.84) | 0.71 (0.35, 1.45) | | 0.98 (0.56, 1.71) |

Adding adjusted for 24-hour potassium excretion groups (<17.4, 17.4 to 24.3, 24.3 to 33.0, ≥28.3 mmol)

Abbreviation: BMI, body mass index.

## Table S7. Association of urinary sodium excretion with incident CVD after adjustment for potential mediators.

| Factor | | Quartiles of urinary sodium excretion, HR (95% CI) | | | |
| --- | --- | --- | --- | --- | --- |
|  |  | Q1 | Q2 | Q3 | Q4 |
| Basic model | | 1 | 1.03 (0.73, 1.47) | 0.92 (0.64, 1.32) | 1.43 (1.02, 1.99) |
| Basic model plus each set of risk factors below added 1 factor at a time | |  |  |  |  |
|  | Carotid Intima-Media Thickness | 1 | 0.97 (0.65, 1.45) | 0.85 (0.57, 1.28) | 1.28 (0.88, 1.86) |
|  | SBP | 1 | 1.12 (0.76, 1.66) | 0.99 (0.66, 1.49) | 1.29 (0.89, 1.87) |
|  | DBP | 1 | 1.10 (0.75, 1.63) | 0.99 (0.66, 1.49) | 1.37 (0.95, 1.99) |
|  | Left ventricular mass | 1 | 1.02 (0.71, 1.46) | 0.88 (0.61, 1.28) | 1.31 (0.93, 1.84) |

Abbreviations: DBP, diastolic blood pressure; SBP, systolic blood pressure.

Basic model was adjusted for age (35–44, 45–54, 55–64, 65–74, ≥75 years of age), sex, body mass index (<18, 18 to 20.9, 21 to 22.9, 23 to 24.9, or ≥25 kg/m^2^), smoking (yes/no or abstinence), current alcohol drinking (regular/no), marital status (single, married and living with a spouse, or divorced and separated), regular exercise habit (yes/no), an education level (less than 9 years, at least 9 years), occupation (no work, labor, official or business), low-density lipoprotein(mg/dL), glomerular filtration rate (<60,≥60 mL/min/1.73 m^2^) and diabetes status (yes/no)

Adjusted models were adjusted for the variables in the basic model plus each of the risk factors added 1 factor at a time to separate models.

The proportion was calculated as (HR_basic model_− HR_adjusted model_) / (HR_basic model_ − 1) × 100%

**Figure S1. Study flow chart of this study**
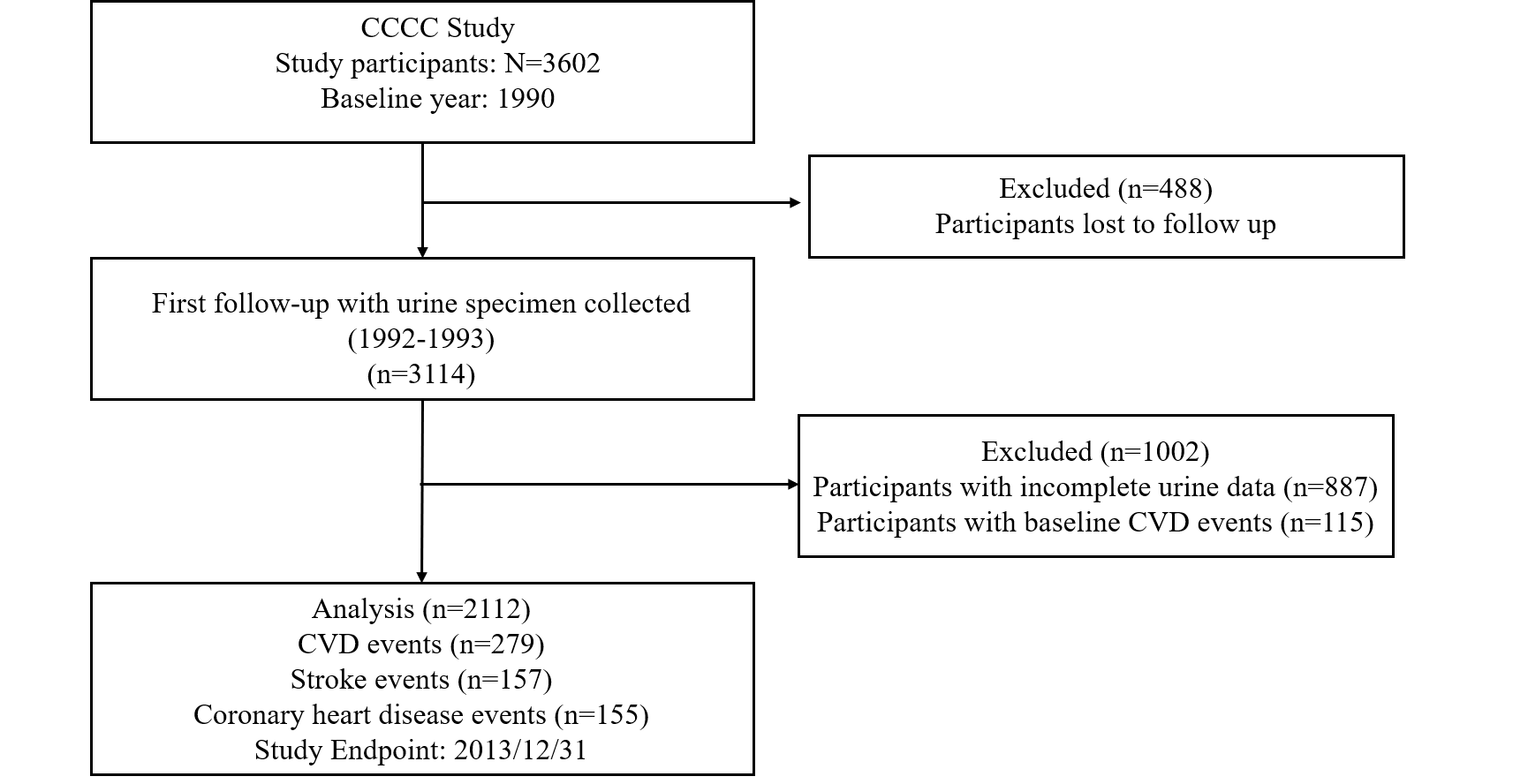


## Figure S2. The linear (A) and non-linear (B) association of urinary sodium excretion and HR of cardiovascular disease.


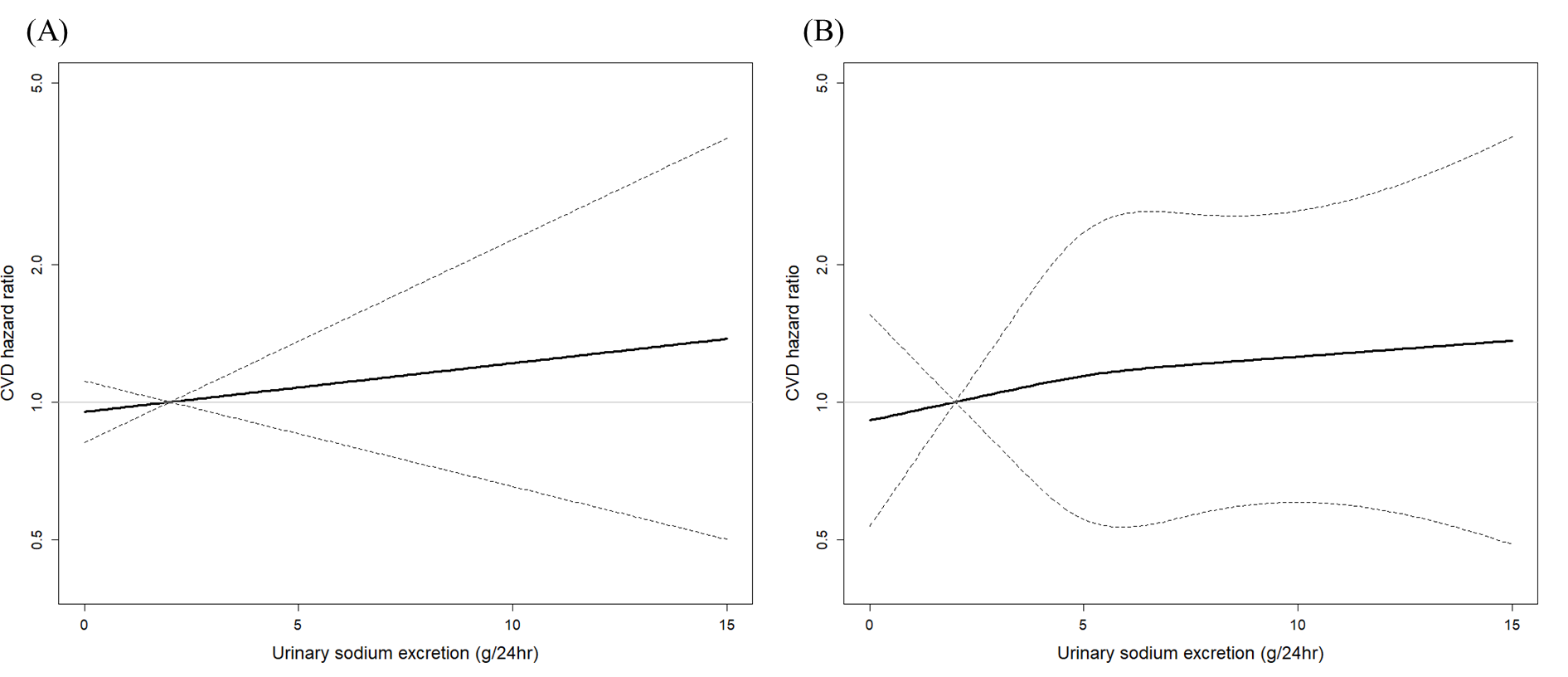


P for the linear model:0.54; P for non-linear model: 0.73; P for ANOVA test:0.87

## Figure S3. The linear (A) and non-linear (B) association of urinary sodium excretion and HR of stroke.


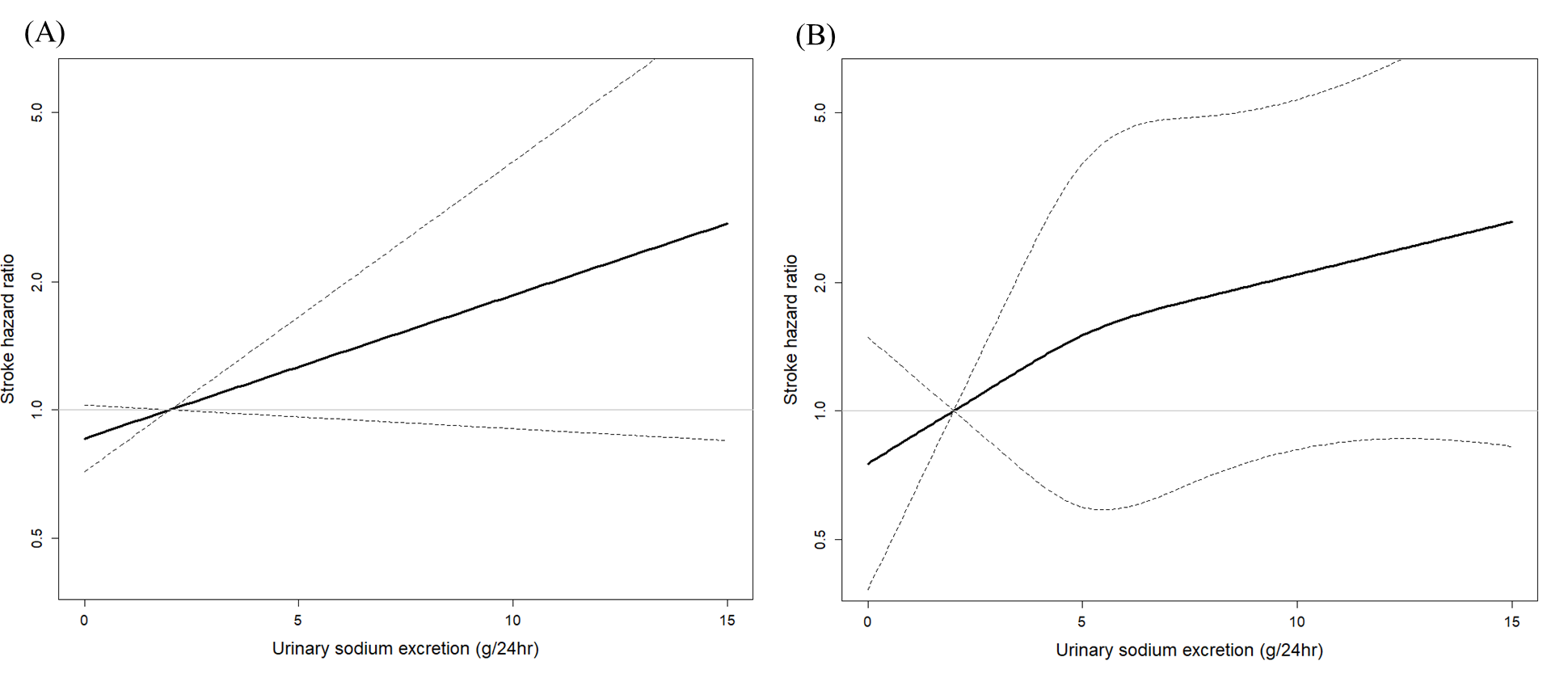


P for the linear model:0.09; P for non-linear model: 0.41; P for ANOVA test:0.70

## Figure S4. The linear (A) and non-linear (B) association of urinary sodium excretion and HR of coronary heart disease.


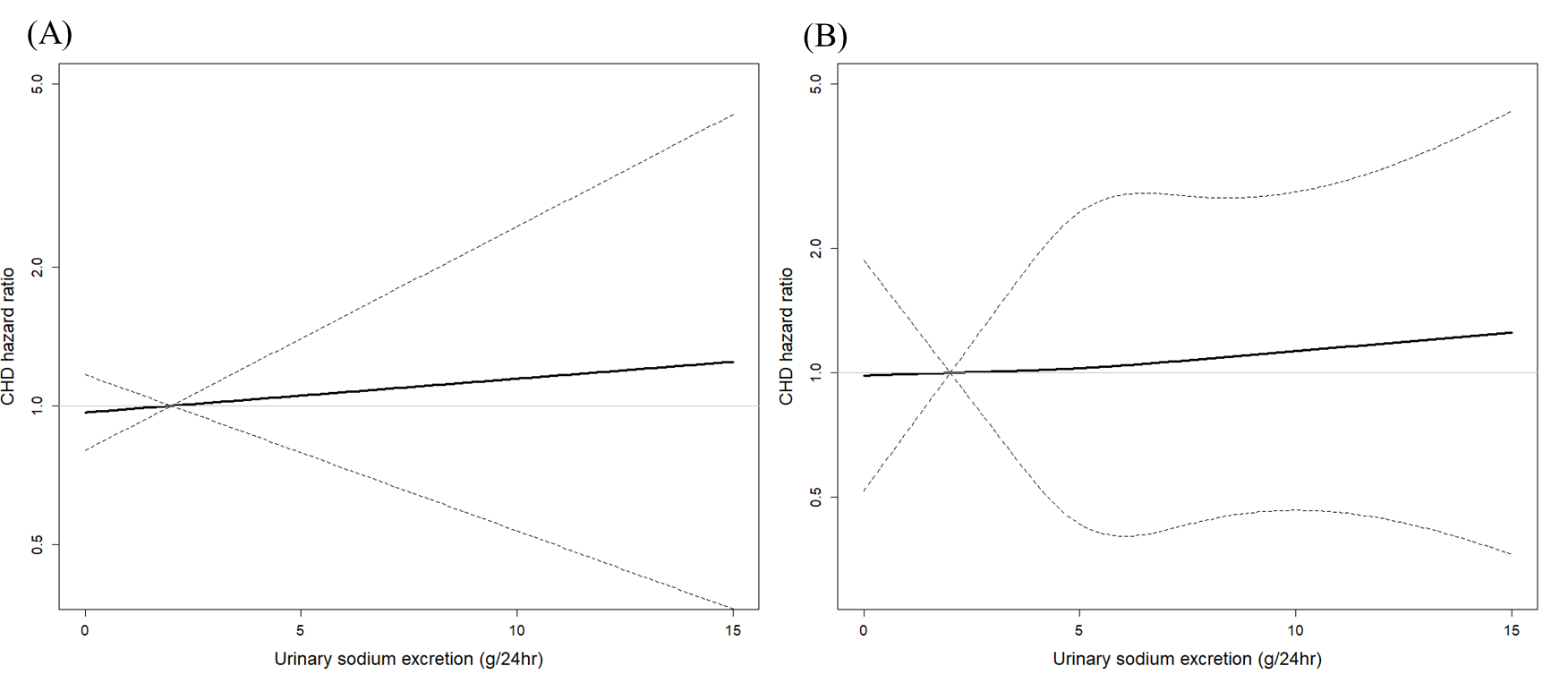


P for the linear model:0.73; P for non-linear model: 0.33; P for ANOVA test:0.96

## Figure S5. The proportion of CVD risk reduction for the highest group of urinary sodium excretion in the counterfactual framework approach.


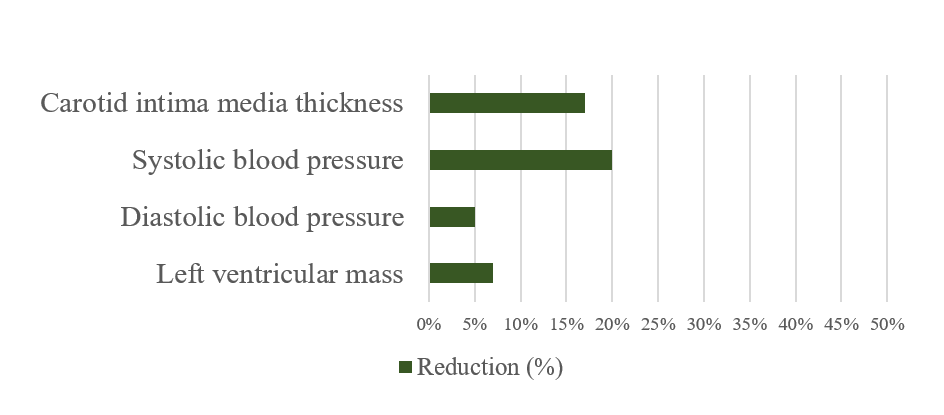


**Figure S6.** **Scatterplots and correlations for overnight urine sodium excretion, estimated 24-hour urine sodium excretion by the Kawasaki formula^(1)^, and estimated 24-hour urine sodium excretion by the Tanaka formula^(2)^.**


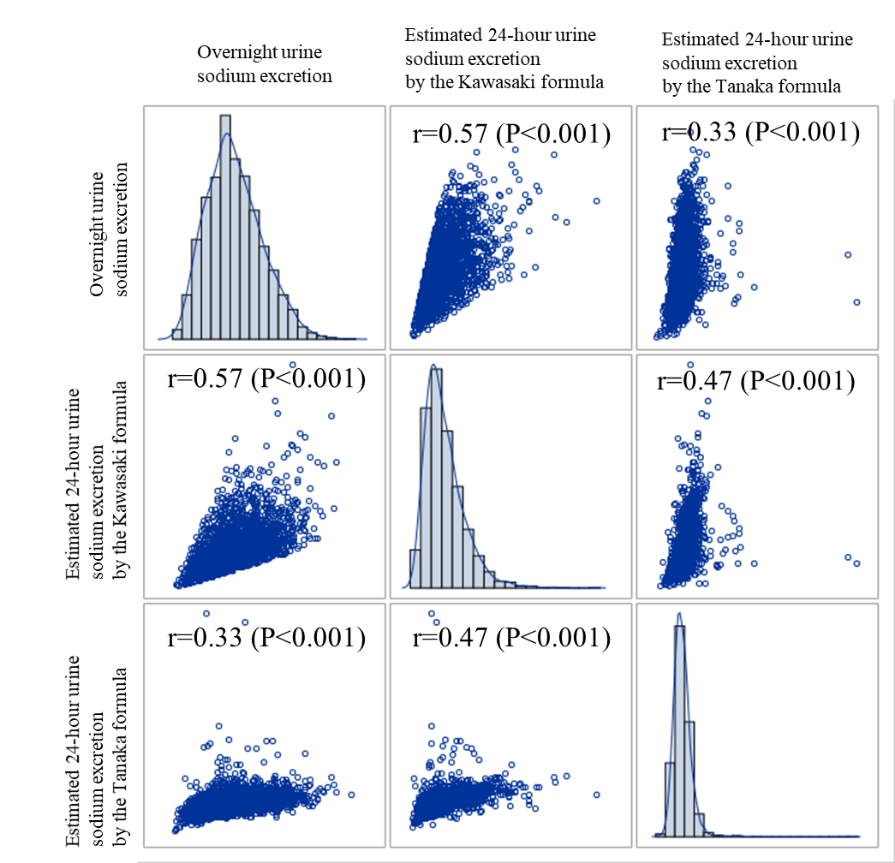


**Reference**

1. Kawasaki T, Itoh K, Uezono K *et al.* (1993) A simple method for estimating 24 h urinary sodium and potassium excretion from second morning voiding urine specimen in adults. *Clin Exp Pharmacol Physiol* 20, 7-14.
2. Tanaka T, Okamura T, Miura K *et al.* (2002) A simple method to estimate populational 24-h urinary sodium and potassium excretion using a casual urine specimen. *Journal of Human Hypertension* 16, 97-103.
